# Orthographic codes in visual word recognition: Task-dependent effects with insertions and deletions of repeated and unique letters

**DOI:** 10.3758/s13421-025-01837-3

**Published:** 2026-02-04

**Authors:** James S. Adelman, Iliyana V. Trifonova

**Affiliations:** 1 Department of Psychology, University of Warwick, Gibbet Hill Road, Coventry, CV4 7AL UK; 2 School of Psychology, University of East Anglia, Norwich Research Park, Norwich, Norfolk, UK; 3 Department of Psychology, University of York, York, YO10 5DD UK

**Keywords:** Word recognition, Orthographic priming, Repeated letters, Lexical decision, Same-different task

## Abstract

How letter position and identity information in strings is processed has been of great importance for visual word recognition and understanding discrimination between similar words. Position and identity are often not a one-to-one mapping, because in most words at least one letter is repeated, occurring in multiple positions. Whether and how multiple correspondences between identity and position affect reading is not yet clear as repeated letter effects occur inconsistently. Here, we investigated this issue with stimuli constructed from words with repetitions (e.g., REJECTS). We manipulated the presence of different identity information by inserting or deleting repeated (e.g., *rejectcs* or *rjects*) or unique letters (e.g., *rejectas* or *rejecs*), either as primes for the base words in lexical decision (Experiment 1) or the same-different task (Experiment 3), or as nonword foils in lexical decision (Experiment 2). The deletion of a repeated letter resulted in shorter response times than the deletion of a unique letter, but only in the primed lexical decision task. In contrast, the insertion of a repeated letter resulted in shorter response times than the insertion of a unique letter, but only for the primed same-different and the unprimed lexical decision tasks. Repetition effects were also observed in the accuracy for the nonword foil rejection for both insertions and deletions. These findings provide further evidence for differential processing between repeated and unique letters, while also showing that absent expected repetitions and present redundant repetitions affect processing according to the task, suggesting task idiosyncrasies in the demands, mechanisms or representations involved.

## Introduction

The essential task of the alphabetic visual word recognition system is to distinguish between strings of letters. It must tell PARKING from PACKING (a difference of identity or substitution), TRAILED from TRIALED (adjacent transposition), WARDENS from WANDERS (non-adjacent transposition), CAPER from CAMPER (unique insertion/deletion) TAPED from TAPPED (adjacent repetition insertion/deletion), and MEDIATE from MEDITATE (non-adjacent repetition insertion/deletion). The key theoretical questions are, of course, about the representations and processes that underpin such discriminations – and the required solutions become progressively more complex as we go through the list of these examples.

Theories focusing on the nature of word form (orthographic) representations and the underlying processes in visual word recognition have been largely based on evidence from studies using the masked-priming paradigm. In this paradigm, a letter string (prime), usually a nonword, is briefly presented between a visual mask (e.g., #####) and the target. Most often, masked priming has been combined with a lexical decision task, in which participants decide whether a written letter string forms a real word or not, and more recently, also with a same-different task, which involves an explicit comparison between two strings. Priming is measured by the difference in response times or accuracy between a condition of interest and a control condition, usually between a form related and an unrelated control prime. Facilitative priming effects suggest that the presentation of the prime has initiated a process in which the target is activated and considered as a possible word candidate. The size of the priming effect has been interpreted in terms of the degree of overlap between the representations of the prime and the target, where stronger primes indicate greater perceptual similarity. Prime manipulations allow for studying how processes such as lexical access and selection occur, and how information related to letter position and letter identity is encoded for the successful discrimination between similar words.

### Outline

Understanding orthographic processing and the processing of letter identity and position would be incomplete if cases in which the same letter identity occurs more than once (i.e., in multiple positions) within words are not also considered. To investigate how processing is affected when some of the constituent letters occupy more than one position within a word, researchers have primarily used deletion and insertion manipulations comparing repeated letters and unique letters. This comparison and these manipulation types are also the focus of the present paper. Here, we explore repeated letter effects with manipulations of insertions and deletions in three different tasks, with the same targets across conditions and tasks. In what follows, we introduce the major findings from the orthographic processing literature with a focus on studies with those manipulations and/or studies focusing on repeated letters. This will be followed by presentation of the computational modelling theory of visual word recognition, and an explanation of how the present experiments are necessary to address both the empirical inconsistencies and the diverging theoretical predictions regarding repetition effects.

### Previous findings

Results from masked primed lexical decision studies including manipulations of letter identity, that is, priming decisions to words like DESIGN with primes like ‘desihn’, ‘dewvgn’ and ‘dzbtkn’ (e.g., Adelman et al., 2014; Lupker & Davis, 2009; Schoonbaert & Grainger, 2004) in which no letter changes position – show that the number of substituted letters affects access to words. Response times are longer following primes with more substitutions. The most effective prime is the one with no letter substitutions, which has the same identity as the target and so is often referred to as an identity prime.

As one might intuitively expect, a variety of results also show that letters that are out of position also influence processing. For instance, words that are anagrams of other words involving minor re-organization of letter order (such as transpositions of adjacent letters) are identified relatively slowly and inaccurately; moreover, such anagrams are often mistaken for words in lexical decision if they are nonwords (e.g., Andrews, 1996; Chambers, 1979; O’Connor & Forster, 1981). The relevant evidence also includes masked primed lexical decision with transposed-letter primes such as ‘jugde’ for JUDGE leading to shorter latencies than primes like ‘jupte’ (e.g., Adelman et al., 2014; Perea & Lupker, 2003) and ‘caniso’ for CASINO versus ‘caviro’ (e.g., Lupker et al., 2008).

Results involving strings created by deletion and insertion have also provided evidence as to the role of letter position when strings do not match in length (e.g., Bowers et al., 2005; Humphreys et al., 1990), again including primed lexical decision with deletion (subset) primes such as ‘degn’ for DESIGN leading to shorter response times than ‘dewvgn’, despite the latter better preserving absolute position of letters (e.g., Adelman et al., 2014; Grainger et al, 2006; Peresotti & Grainger, 1999). Similar studies with insertion (or superset) primes (e.g., ‘desrign’) have found insertion primes to be less effective than identity primes but more effective than substitution primes (e.g., Adelman et al., 2014; Lupker, Zhang et al., 2015b; Van Assche & Grainger, 2006; Welvaert et al., 2008).

Turning to our key issue of repeated letters, Schoonbaert and Grainger (2004) compared deletion primes that did or did not affect the repetition in words with repeated letters, such as ‘balnce’ or ‘balace’ for BALANCE, and found no difference in priming, and thus no evidence that the representation of a prime that retains the repetition is more or less similar to the target’s representation than the prime that disrupts the repetition and retains instead an additional unique letter. However, the target words with repetitions received slower responses than matched target words without repetitions, suggesting that words with repetitions were more difficult, albeit this difference was only significant in the by-subjects analysis. They also used both types of deletion primes as nonword targets in unprimed lexical decision, and the results showed no repetition effect in either response time or accuracy.

Instead of deletion primes, Van Assche and Grainger (2006) compared insertion primes that created a repetition, such as ‘jusstice’ for JUSTICE, and insertion primes that did not create a repetition, such as ‘juastice’, and again found no difference in priming, both for adjacent and non-adjacent repetitions, even with stronger manipulations affecting more letters, despite all insertion primes being less effective than the identity prime. Thus, the masked-priming lexical decision results with both insertion and deletion did not provide any evidence for differential processing between repeated and unique letters.

Norris et al. (2010) argued that the non-significant differences of the prior studies were not strong evidence against models that might predict repeated-letter effects because there may only be a very weak effect; there could be greater power to detect a repeated-letter effect with a stronger manipulation and a different task. They used prime-target combinations such as ‘uueer’ for UNDER to be compared with ‘ulger’ that used double substitutions rather than insertions, and in which the letter repetitions were adjacent. They also included deletion manipulations in stimuli such as ‘anex’ for ANNEX to be compared with ‘eupt’ for ERUPT, with the identity priming condition (rather than an all-letter-different condition) as the control. Across their experiments, they also compared the primed lexical decision task with the primed same-different task. In the latter task, a reference is presented clearly before the (brief) prime and target, with the reference and target to be compared. Priming on same trials is typically larger than that for word trials in lexical decision (Kinoshita & Norris, 2009; Norris & Kinoshita, 2008). Indeed, for the substitutions, the same-different task showed a significant response-time advantage for the repeated-letter condition, whereas the lexical decision task showed a smaller, non-significant effect. In primed same-different (Experiment 3a) and primed lexical decision (Experiment 3b) tasks, the difference between identity and deletion primes was significant only for the deletion of a unique letter from an ERUPT-like stimulus, not a repeated letter from an ANNEX-like stimulus. However, the interaction between target type and the contrast between prime types deleting unique or repeated letters was not itself significant. Nevertheless, Norris et al. interpreted the pattern of one significant and one non-significant effect as evidence for a repetition effect.

Adelman et al. (2014) ran a large study of primed lexical decision with many distinct prime types. Of these, only one related to repeated letters, which used a prime that inserted two repeated but unrelated letters (e.g., deshhign-DESIGN); this led to significantly shorter response times than the corresponding insertion of two distinct letters (e.g., desaxign-DESIGN). This differs from the other studies considered here, and the focus of the present paper, however, in that the repetition does not involve any letters of the target word.

Gomez et al. (2008) investigated both adjacent and non-adjacent repetitions, using two-alternative forced-choice identification of briefly presented nonword strings. In this paradigm, participants choose between two items (target and foil) after the brief presentation of the target and lower accuracy indicates stronger similarity between the target and the foil. In their Experiment 4, Gomez et al. included:

(i) Foil-repetition items in which a target with no repeated letter (ABCDE) was probed with a foil with a letter repetition in substitution of a correct letter (either adjacent: e.g., ABCDE [correct] vs. ABBDE; or non-adjacent, e.g., ABCDE [correct] vs. ABCBE);
(ii) Target-repetition items in which a target with a repetition (adjacent: e.g., ABBDE or non-adjacent: ABCBE) was probed with foil that replaced an instance of the repeated letter with a unique letter (e.g., ABBDE [correct] vs. ABCDE; or ABCBE [correct] vs. ABCDE);
(iii) Both-repetition items where both items shared the same number of each letter identity and the same distance between the repetition (e.g., ABBDE vs. ADBBE; or ABCBE vs. ACBEB);
(iv) Transposition items; and
(v) Substitution controls in which unique substitutions were made to targets with no repetition (ABCDE vs. ABXDE).

Overall, accuracy was higher when targets with no repeated letters were paired with foils with repetitions than when targets with repetitions (both adjacent and non-adjacent) were paired with foils with no repeated letters. When the target had no repetition, accuracy was similar in rejecting foils that substitute a repeated letter adjacent to the original to those than substitute a novel letter, but accuracy was higher if the foil substituted a repeated letter non-adjacent to the original. Performance for trials where both target and foil contained repetitions depended on the position of affected letters: Accuracy was higher than controls for trials in which the identity of the initial letter differed between options and for items in which one option had an adjacent final-repetition and the other had that repetition in a different position. Adjacent both-repetition items manipulating only internal letters had performance similar to controls. Non-adjacent both-repetition items that did not affect the initial letter had worse performance than controls. Most importantly, the study of Gomez et al. provided some evidence suggesting that repeated letters might be harder to identify than unique ones for both adjacent and non-adjacent cases.

Trifonova and Adelman (2022) conducted a series of experiments investigating non-adjacent letter repetitions. In their first experiment, like Gomez et al. (2008), Trifonova and Adelman used two-alternative forced choice identification of briefly presented strings, but their manipulations used insertions and deletions rather than substitutions. The results showed that foils created by inserting unique letters (e.g., DRALTIEN from DRATIEN) were more accurately rejected than those created by inserting repeated letters (e.g., DRARTIEN from DRATIEN), whereas there was no difference for deletions created by deleting a repetition or a unique letter (with the roles of the stimuli reversed). In subsequent experiments with the primed same-different task with stimuli from the same set, priming from deletions (relative to a less-related control) did not differ depending on repetition, but responses were faster following a prime that had a repeated letter inserted than those that had a unique letter inserted. The result from the insertion primes, therefore, contradicted previous masked-priming studies showing no difference between repeated and unique letter insertion primes (Van Assche & Grainger, 2006) and provided evidence for non-adjacent repeated letter effects with the masked-priming paradigm when combined with the same-different task. The stronger priming from the repeated letter primes than the unique letter primes suggested that repetition primes were more similar to the target and the inconsistency coming from a redundant repeated letter is less disruptive to the processing of the target than the inconsistency coming from a redundant different letter. Trifonova and Adelman explained this result in terms of higher processing demands for the detection of inconsistency in the case of repeated letters than unique letters, and suggested that the precise processing of repetitions, in terms of identity as well as position, could be harder than that of unique letters.

Results from unprimed word recognition also point towards differences between repetitions and unique letter identities. Trifonova and Adelman (2019) examined response times to words in English (Balota et al., 2007), British (Keuleers et al., 2012), French (Ferrand et al., 2010) and Second Dutch (Brysbaert et al., 2016) Lexicon Projects using regression analyses, and found that non-adjacent repetitions were associated with longer response times, similar to the result for targets of Schoonbaert and Grainger’s (2004) Experiment 1. These results suggested that words containing such repetitions were harder to process. Kerr et al. (2021) constructed nonwords for lexical decision by inserting repeated or unique letters into real words and found slower and less accurate rejections for nonwords where the insertion was a repetition than where the insertion was a novel letter.

In summary, in primed lexical decision, comparisons between manipulations of repeated and unique letters of targets have tended to show more facilitation if repeated letters are manipulated but the relevant comparisons have not reached significance, for deletions (Schoonbaert & Grainger, 2004; see also Norris et al., 2010, n.b., however, that the relevant interaction was not significant in the latter), insertions (Van Assche & Grainger, 2006), or double substitutions (Norris et al., 2010). Therefore, masked priming results with lexical decisions have so far failed to provide evidence for any repeated letter effects.

In primed same-different, primes with double substitutions have shown significantly greater facilitation for word targets when the substitutions repeat existing letters than when they are unique (Norris et al., 2010), and primes with insertions have shown greater facilitation for nonword targets when a repetition is inserted compared with a unique letter (Trifonova & Adelman, 2022). However, contrasts comparing primes that deleted a repeated letter to those deleting a unique letter have not been significant for adjacent repetitions in words (Norris et al., 2010) or non-adjacent repetitions in nonwords (Trifonova & Adelman, 2022). Therefore, masked priming results with same-different decisions have shown repeated letter effects for adjacent letter substitutions with words and non-adjacent insertions with nonwords, but have failed to provide such evidence with deletion manipulations.

In forced-choice identification of briefly presented strings, the interpretation is complicated by suggestions of response bias discussed below. For deletions/insertions, decisions in which one response option contains a repetition have lower discriminability than those where neither response option has a repetition (Trifonova & Adelman, 2022; Experiment 1), but this was only evident for insertion foils. For substitutions, unique-letter foils for repeated-letter targets yielded lower accuracy than repeated-letter foils for unique-letter targets, and unique-letter foils for unique-letter targets were overall intermediate (Gomez et al., 2008, Experiment 4).

In unprimed lexical decision, word targets with repeated letters (at least non-adjacent ones) tend to have longer response times (Trifonova & Adelman, 2019). Nonword targets that have insertions relative to basewords are more difficult to reject if those insertions are repetitions (Kerr et al., 2021), but there is no corresponding significant result for deletions.

Broadly speaking, there is evidence that there are at least some effects involving repeated letters, though little of it comes from primed lexical decision, the primary paradigm in the field. It has been suggested that the non-significant effects in primed lexical decision reflect low power for small effects by those proposing models predicting such effects (e.g., Adelman, 2011; Norris et al., 2010). We now consider models relevant to the role of repeated letters in the identification of words and other letter strings.

### Models

Many early and influential models of visual word recognition were developed for the processing of strings of fixed length (in letters) and contained simplifying assumptions that each letter was processed independently at the lower perceptual levels in a fixed ordinal position (e.g., McClelland & Rumelhart, 1981; Rumelhart & Siple, 1974). In these models, comparison of the stimulus to response options or lexical entries is based on comparing each perceived letter with letters in the corresponding positions in lexical entries and/or response options. Models based on this approach could give some account of priming effects based on numbers of substitutions but could not speak to other effects involving transposed letters or strings of different lengths.

Subsequent models of the orthographic input coding system (e.g., Adelman, 2011; Davis, 1999, 2010; Gomez et al, 2008; Grainger & van Heuven, 2003; Norris et al., 2010; Whitney, 2001) have provided ways to deal with positional information about letters in an effort that has been labelled ‘cracking the orthographic code’ (Grainger, 2008). These – directly or indirectly – offer possibilities for the representation and processing of repeated letters as compared with unique letters, and how these representations and processes will produce effects (or not) of manipulations involving insertion or deletion.

#### Open bigram frameworks

One such approach is the open bigram framework, as in the models of Whitney (2001) and Grainger and van Heuven (2003). In these models, units are activated for ordered pairs of letters that need not be adjacent, such as CA, CT and AT for CAT. If all consistent open bigrams were considered – an unconstrained open-bigram scheme – then the amount of priming in primed lexical decision would be determined by the number of the open bigrams activated by the prime that are among the bigrams that would be activated by the target (if inhibition is neglected; see below). Deleting a repeated letter removes fewer unique bigrams than deleting a unique letter, so a repeated letter effect would be predicted for deletion priming and similar experiments. However, adding a letter to the identity prime does not change the number of consistent bigrams – regardless of whether the added letter is a repetition or unique, all target bigrams are active. Therefore, no difference can be predicted between repeated and unique insertions in insertion priming and similar paradigms – indeed, they should be the same as identity primes. Finally, if we consider the comparison between targets with and without repeated letters, then there are fewer unique open bigrams in target with repeated letters because there are duplicates; this could lead to slower responding to these targets.

The model proposed by Grainger and van Heuven (2003) was not, however, this kind of unconstrained open-bigram model. As an approximation to the idea that activation should be weaker for more distant bigrams (borrowed from Whitney’s model) they constrained the open bigrams, so that bigrams with a distance of more than two intervening letters are not represented. In this representational format, insertion of a letter makes some bigrams too distant to appear in the prime representation, but if a repetition is inserted this re-creates some of the bigrams that would be lost by inserting a unique letter. As such, there is more overlap with the target for the repeated insertion than the unique insertion, predicting greater priming for repeated insertion primes. Schoonbaert and Grainger (2004) argued that no difference was predicted by the Grainger and van Heuven scheme between deletions of unique and repeated letters based on the examples balnce-BALANCE having ten matching bigrams BA, BL, AL, AN, AC, LN, LC, NC, NE and CE, and balace-BALANCE having ten matching bigrams BA, BL, AL, AA, AC, LA, LC, AC, AE and CE.^1^ A later, graded version of the bigram model (Grainger et al., 2006), in which the model attempts to code only closed (i.e., adjacent) bigrams, but positional uncertainty results in partial activation of more distant and reversed bigrams, also predicts repetition effects for deletion primes.

Turning to the cases where letters are inserted, Van Assche and Grainger (2006) observed that for their stimuli, constrained open-bigram schemes predict that inserting a repeated letter preserves more consistent bigrams than inserting a unique letter, so the repetition-insertion prime is predicted to give more facilitation than the unique-insertion prime.^2^

In summary, open bigram schemes suggest that words containing repeated letters might be harder to access due to having fewer unique sublexical representations supporting their activation (in this case, open bigrams) than targets with the same length and no repeated letters (as duplicated bigrams are not included). Moreover, primes that manipulate (delete or insert) repeated letters should be more effective (facilitatory) than those that manipulate unique letters because they disrupt fewer consistent bigrams and/or create fewer inconsistent bigrams.

#### Spatial coding

Davis’s (1999, 2010) models include a spatial coding mechanism that assigns each repetition of a letter to a separate copy of the relevant letter channel, so that repeated and unique letters have the same overall role in the spatial coding calculation that determines the input to word nodes. In this model, each lexical entry has a receiver node for each of its constituent letters, separately for repeated letters. Each receiver can receive input from only one perceived letter (or none) and each perceived letter can feed only one receiver (or it adds to the inhibitory mismatch term if it is surplus). As no letter can do ‘double duty’, repeated letters are not functionally different from unique ones, and no repetition effects can be predicted for primed tasks or unprimed lexical decision.

Observations of repetition effects suggest that the mechanism used by Davis’s (2010) model to separate repeated letters into different channels is not a good account of how repeated letters are cognitively processed;^3^ it is possible to imagine different versions of the superposition system the model uses that instead use one channel per letter identity, but the consequences would require simulation.

#### Leakage or sloppy slots models

In the models of Gomez et al. (2008) and Norris et al. (2010), positional uncertainty is implemented as a distribution so that evidence from a letter in one position can ‘leak’ into nearby positions. There is no separation of channels to prevent a single letter in the stimulus contributing to evidence for two letters that are repeated near each other in a lexical entry (or other string) nor to prevent two copies of a letter in the stimulus both contributing to evidence for the same instance of a letter in a lexical entry (or other string). From these distributions, Gomez et al.’s model is designed for two-alternative forced-choice identification of a briefly presented stimulus (as in their experiments described above). The model uses a (deterministic) perceptual distribution generated by the target stimulus to compute overlap scores between that distribution and the options in the forced choice; these scores are raised to a power before being entered into the Luce (1959) choice rule to determine the accuracy of responses. The model was successful across a range of experiments (not only those including repeated letters described above). However, to accommodate the observed repeated-letter data, the power parameter that was applied to the overlap score for each response option in the forced choice depended on whether the option contained a repeated letter; with the fitted values, this resulted in decisions being biased against the option with repeated letters (which could be the target or the foil).

Gomez et al. (2008) also sketched out how their overlap scores might be used to generalize to priming paradigms. Its predictions are therefore consistent with the result for insertions, but also appear somewhat inconsistent with no repetition effect for deletions. However, the interpretation for deletions is complicated by the fact that the targets for the repeated deletion prime condition in some studies had higher self-similarity (due to leakage) than did the targets for the unique deletion prime condition. If extended to word target effects in lexical decision, this might imply that targets with repetitions might be easier to respond to – because they are more self-similar – contrary to the observed effects in response time.

Norris et al.’s (2010) model draws random perceptual samples from the stimulus that are evaluated against likelihood functions representing possible stimuli (such as words in the vocabulary) that allow for positional leakage. As more samples are taken, the likelihoods change, tending to more strongly favor the presented stimulus over time, allowing response time predictions to be made, including for primed lexical decision and same-different tasks. The allowance for leakage in the likelihood function makes the repeated-letter primes (e.g., ‘uueer’) in Norris et al.’s double substitution experiments more facilitatory (for, e.g., ‘UNDER’) than the unique-letter primes (e.g., ‘ulger’) according to the model’s predictions, consistent with how Norris et al. interpreted their (Experiment 2) same-different task result (treating a non-significant interaction as significant). This occurs because both U1 and U2 of ‘uueer’ provide evidence toward U1 of ‘UNDER’ (and E3 and E4 towards E4).

Norris et al.’s (2010) model was designed only for stimuli of fixed length and was not extended to account for their final experiment in which deletion primes were shorter than targets differed in length. Instead, they gave a verbal argument that the principles of leakage meant that their model would predict a repetition effect in deletion conditions.^4^

The fact that leakage makes items with repeated letters more similar to themselves not only complicates the interpretation of repeated-letter effects with a between-target manipulation, but would normally imply that response times should be shorter to repeated-letter targets in lexical decision, though the opposite is generally found empirically.

#### LTRS

In Adelman’s (2011) LTRS model, the perceptual representation changes over time in a different fashion: The percept is built up in a piecemeal random fashion. Incomplete percepts are compatible with more possibilities than just the target. In forced choice identification, the incomplete percept is compared directly to the response options, and if the percept is compatible with both, a guess must be made. Therefore, accuracy depends on whether the target percept can be distinguished from the foil after target offset. In priming, many potential targets are primed to an amount equal to how long the target percept is compatible with each target: Facilitation depends on how long the prime percept cannot be distinguished from the pre-established target representation (e.g., lexical entry). The consequences for insertion primes are that an inserted repeated letter can sometimes masquerade as its twin in the incomplete percept, so it does not immediately provide evidence against the target word, whereas a novel inserted letter is immediately out of place, providing evidence against the target word; for instance, perceiving the third letter of ‘jusstice’ does not provide evidence against ‘JUSTICE’ unless the fourth letter is also perceived, but the third letter of ‘juastice’ unambiguously provides evidence against ‘JUSTICE’. As such, repetition effects are predicted: lower accuracy for repeated-insertion foils than unique-insertion foils; and greater facilitation for repeated-insertion primes than unique-insertion primes.

As such, Adelman (2011) suggested that the null effect of Van Assche and Grainger (2006) for the comparison of repeated and unique insertions was essentially a Type II error, given that LTRS predicts a 10-ms effect when a 2-ms effect was observed, and Van Assche and Grainger’s other 10-ms effects did not reach significance. Not only must there be limited power to detect an effect of the predicted magnitude, but it also implies that 10ms would be within the confidence interval on the estimate of the raw effect size.

LTRS has no analogous process that can affect priming when the repetition is only in the target – processing is the same regardless of whether a letter in the stimulus matches one letter in a lexical entry or two, because the model gives no possibility for information to be missing from the lexical entry.^5^ Regardless of whether the letter removed from the target to produce the prime is repeated (e.g., blance) or not (e.g., balnce), absence of perceptual evidence is not evidence of absence: The letter might just not have been perceived yet. For percepts from deletion primes to be incompatible with the target, the incorrect adjacency information (or closed bigram: bl in blance or ln in balnce) must be perceived, the timing of which (for matched positions) does not differ on the basis of a letter not in the prime.

LTRS predictions for both insertion and deletion primes were consistent with Trifonova and Adelman’s (2022) Experiments 2 and 3 with the same-different task: a repetition effect only for insertions. For forced-choice identification, the model was generally successful in modeling Gomez et al.’s (2008) data. For Trifonova and Adelman’s Experiment 1 with forced-choice identification, though, the base LTRS model predicted a repetition effect for deletion foils only (where the manipulation affects the information that can be perceived in the target), contrary to the finding that there was a repetition effect only for insertion foils. However, a simple change to the guessing rule – to more often choose response options with repeated letters than those without – allowed the model to make the correct prediction. LTRS only models the processing and consequences of briefly presented stimuli, without a full lexical decision model, so cannot address differences between repeated and unique targets.

### Motivation for the present studies

Overall, there are limitations in what the existing evidence about repeated letters can tell us about the models that do predict repetition effects. It is unclear whether there are real asymmetries between insertion and deletion because many of the comparisons are cross-experiment and/or involve the combination of two statistical results, rather than a single significant interaction, which is related to the potential lack of power in some studies. These problems are exacerbated in several respects by priming different targets for deletions of repetitions and deletions of unique letters. First, the insertions and deletions are not directly comparable. Second, the predictions of coding schemes in the absence of a process model can be hard to derive when comparing across target representations. Finally, when using different targets, the choice of control condition becomes important, and one is looking for a (less powerful) interaction, rather than comparing the two conditions of interest (repeated vs. unique letter) directly. Moreover, despite some advantages of the same-different task, the primed lexical decision task remains the primary mode of investigation, and the one that most models are geared toward. The argument that the same-different task more directly taps front-end processes of visual word processing is a mixed blessing: That the same-different task is orthography- and stimulus-driven suggests that the prime and target are being compared to a short-term memorial representation of the stimulus. The nature of this process and representation could be quite different from that involved in the attempt to access a lexical entry from a stimulus, which is relevant to reading, and one would hope and assume, priming when the task requires lexical access, as in primed lexical decision. A systematic investigation of repeated letter effects is therefore necessary, in which the target word remains the same across conditions and contains repeated letters. As some evidence suggests that words with repetitions are more difficult to access, for example, due to fewer sublexical representations that could activate them, manipulations aiming to investigate the effect of the presence or absence of repeated or unique letters should remain within-target to eliminate additional complexity in the interpretation of the results. Deletion and insertion manipulations are an important test for the role of repeated and unique letter identities in word processing, as theories are not unanimous in terms of their repeated letter predictions, with some models predicting differential processing for repetitions in cases of insertions but not deletions, or different size of the repeated letter effects with these two manipulations. Another important question would be if the masked-priming findings of the lexical decision task and the same-different task would converge, or whether there would be different susceptibility to the repeated letter effect within these manipulations due to the processing focus of these tasks.

The use of nonword rejections in unprimed lexical decision can also be useful, but since the nonwords on the trials of interest are very word-like, rejections likely depend on an additional controlled process that checks the spelling of the target against the retrieved lexical representation in addition to the access process that priming is usually used to investigate. It appears that while it is available to readers, this spell-checking process is likely rarely used, given how frequently readers are unaware of spelling mistakes (even when they slow reading down). This type of spell-checking process has been frequently hypothesized but rarely modeled.

### Overview of experiments

The present work was therefore intended to check if the asymmetry observed by Trifonova and Adelman (2022) in the primed same-different task – repetition effects for insertions not deletions – generalized to words and to the lexical decision task. We aimed to make the results more interpretable by using the same targets across all conditions and experiments. We aimed to make the experiments more powerful than most prior experiments by using more participants and targets and by focusing our design on four core conditions of interest, crossing repeated and unique letters with insertions and deletions.

All three experiments were based on the same stimuli constructed from seven- to nine-letter base words containing a repeated letter pair with one intervening letter (e.g., REJECTS; MEANING). These stimuli inserted a letter that formed another repetition (e.g., rejectcs; maeaning) or a letter that would be unique (rejectas; mceaning) or deleted a repeated letter (e.g., rjects; meanig) or unique letter (rejecs; maning) from the base word. In Experiment 1, the base words were lexical decision targets, primed by the four types of constructed stimuli. In Experiment 2, the constructed stimuli were targets (requiring a negative response) in an unprimed lexical decision task; the base words were not presented. In Experiment 3, the base words were used as the reference and target on the same trials of a same-different task and the constructed stimuli were used as primes. Table 1 compares the numbers of subjects and trials per condition per participant of the current studies and the most relevant prior studies. Only the more recent studies have over 1,000 total trials per condition, with most having over the 1,600 advocated by Brysbaert and Stevens (2018); the exception, Trifonova and Adelman’s (2022) Experiment 2, had 1,408, and the non-significant repetition effect there was not in the expected direction.

**Table 1:** Comparison of data sets in prior experiments comparing repeated and unique letters using manipulations of insertions, deletions and/or substitutions

Experiment(s)	Subjects	Trials per subject per condition	Task	Key relevant manipulation
Schoonbaert and Grainger (2004) Experiments 1 and 2	44, 40	15	primed LDT, LDT (rejections)	Primes/nonwords constructed by deletion of a unique or repeated letter from a word.
Van Assche and Grainger (2006) Experiments 1a and 1b	40 (20 & 20)	15	primed LDT	Primes inserting a unique letter or repeating an adjacent letter.
Van Assche and Grainger (2006) Experiments 2a and 2b	36 (18 & 18)	15	primed LDT	Primes inserting two repetitions of the same adjacent letter or two unique letters.
Norris et al. (2010) Experiment 1	25	20	primed LDT	Primes replacing two letters with unique letters or with repetitions of different adjacent letters.
Norris et al. (2010) Experiments 2a and 2b	25, 25	20	primed same-diff.	Primes replacing two letters with unique letters or with repetitions of different adjacent letters.
Norris et al. (2010) Experiments 3a and 3b	30, 30	20	primed same-diff., primed LDT	Primes with deletion of a unique or repeated letter.
Trifonova and Adelman (2022) Experiment 1	96	88	2AFC identification	Foils created by deletion or insertion of a unique or non-adjacent repeated letter.
Trifonova and Adelman (2022) Experiments 2 and 3	64, 72	22, 58	primed same-diff.,primed same-diff.	Primes created by deletion or insertion of a unique or non-adjacent repeated letter.
Kerr et al. (2021)	36, 40	50, 25	LDT (rejections)	Nonwords constructed by insertion of a unique or non-adjacent repeated letter to a base word
Current Experiments 1, 2 and 3	80, 88, 72	36	primed LDT, LDT (rejections) primed same-diff.	Primes/nonwords created by deletion or insertion of a unique or non-adjacent repeated letter of a word.

*Note:* Studies are listed in chronological order of data collection rather than eventual publication

The stimuli used across all three experiments are detailed below; additional stimuli are described for each experiment in the relevant Methods. All three experiments fall within the University of Warwick Humanities and Social Sciences Ethics Committee approval HSS 62/18–19.

### Transparency and openness

All lists of stimuli, experimental scripts and analysis scripts are available on the Open Science Framework (OSF) at: https://osf.io/a43h7/. No pre-registrations were made for these experiments.

### Common stimuli of interest

#### Base words

Base words (total: 144) that were seven letters long (76), eight letters long (50) or nine letters long (18) were chosen from the British Lexicon Project that had accuracy in excess of 85% in that database. All contained a single letter repetition separated by a single letter; the repetition did not involve either exterior letter. The base words were paired so that the repetitions did not occur in the same positions in the two words. Base words were re-selected and re-paired in a random trial-and-error process to attempt to create as many stimuli as possible in which the stimuli created by the below procedures did not contain any unintended repetitions of letters.

#### Nonwords

Nonwords were constructed by deletion and by insertion for use as primes, or for use as targets in unprimed lexical decision. Due to an oversight, three stimuli intended to be nonwords were words; this error was noted only after Experiment 1 was complete. These erroneous stimuli were retained for Experiments 2 and 3, but data associated with these stimuli were excluded from analysis.^6^

#### By deletion

From each base word, two types of deletion nonword were constructed. The first was created by deleting the repeated letter that was further from the center of the word, such as ‘rjects’ from ‘rejects’ and ‘meanig’ from ‘meaning’. The second was created by deleting a unique letter that was in the position being deleted for the repetition in the other base word of the pair. This process matched the positions of deletions across the two conditions: to balance ‘rjects’ constructed by deleting a repeated ‘e’ from ‘rejects’, ‘maning’ was constructed by deleting a unique ‘e’ from ‘meaning’, both from position 2. Similarly, ‘rejecs’ deleting a ‘t’ balanced ‘meanig’ deleting an ‘n’, both from position 6.

#### By insertion

From each base word, two types of insertion nonword were constructed. The first added a second repetition, by repeating the letter one position more central than that involved in the unique deletion condition; the letter was adding two positions away from the center. Thus, from ‘rejects’, for which ‘t’ was deleted, ‘rejectcs’ was constructed, by making a copy of the ‘t’’s inside neighbor (‘c’) outside it. Similarly, for ‘meaning’, the ‘e’ was flanked by an additional copy of its inside neighbor (‘a’) to create ‘maeaning’. The position of insertion of new unique letters was the same as for the repetition with the same target (i.e., was not based on the partner) and the identity of the unique insertion was matched within a pair: ‘a’ was the unique insertion for ‘rejects’ to create ‘rejectas’ (the identity from ‘maeaning’ and the position from ‘rejectcs’) and ‘c’ was the unique insertion for ‘meaning’ to create ‘mceaning’. Thus, the number of ‘a’s (or ‘b’s etc. or vowels or consonants) inserted in the unique condition was the same as in the repeated condition.

#### Neighborhood properties of the stimuli

The average OLD20 (Yarkoni et al., 2008) of the base words, based on words in SUBTLEX-UK tagged as correct UK spellings, was 2.19 (*SD* 0.48).

For nonwords constructed by deletion of a repeated letter it was 2.14 (*SD* 0.37) and for unique deletions 2.18 (*SD* 0.40), which did not differ significantly, paired *t*(143) = 1.31, *p* =.192. For those constructed by inserting a repetition it was 2.96 (*SD* 0.45) and for unique insertions 2.92 (*SD* 0.46); though of similar magnitude to the difference for deletions, this difference was significant, paired *t*(143) = 2.66, *p* =.009, because the correlation between the OLD20s of the insertion pairs was stronger than that for the deletion pairs (*r* =.938 vs..541), which increases the power of the paired *t*-test. The OLD20 was higher for insertions because the insertions usually produced an orthotactically illegal or rare sequence that was essentially only resolved by deleting that letter, whereas for deletions, orthotactically illegal sequences were less frequent, and when they did occur, they could potentially be resolved by an insertion of more than one different letter.^7^

## Experiment 1

Experiment 1 was the primed lexical decision experiment. We examined response times to words following primes with inserted or deleted letters that were either repetitions or unique.

### Method

#### Participants

One hundred and thirty-two first-year undergraduates participated for partial course credit. Data of 35 non-native speakers of English were removed as were those of seven other participants with accuracy below 85%. One counterbalancing list had remaining data for only 20 participants, so data of ten further participants were removed from other lists to bring them to this number, which were the latest collected for their list. Data of 80 participants were therefore analysed.

Experiments 1 and 2 have largely completely overlapping participant sets because both were offered to the same cohort; most completed Experiment 1, which was in the laboratory, before Experiment 2 was made available online.^8^

#### Stimuli

For the word trials, the primes were the constructed nonwords and the targets were the base words described in the Common Stimuli of Interest above.

For the lexical decision foils, nonwords were selected from the British Lexicon Project (Keuleers et al., 2012) in the same numbers of each length (in letters) as the target words. All had a repeated letter with a single intervening other letter, and an observed accuracy of at least 85%. All four prime types were constructed for each nonword target by insertion and deletion of repeated and unique letters. Nonword targets were not paired with other targets by selection position, and the choice of unique insertion letters was random.

#### Counterbalancing lists

Four lists were constructed that each had a quarter of trials with each prime type, equally for word and nonword targets. Across lists, every prime-target combination appeared exactly once.

#### Procedure

Participants were instructed that their task was to examine the upper-case letter string on each trial and press the right shift key if it was a real English word or the left shift key if it was not. Following 12 practice trials, all trials from the relevant counterbalancing list were presented in a new random order.

On each trial, DMDX (Forster & Forster, 2003) displayed a ######### mask for 500 ms, then the 12.5 pt Courier New lower-case prime for 50 ms, then the 20 pt upper-case target until response or a maximum of 3,000 ms had elapsed. Feedback was given after incorrect responses and timeouts only.

### Results

#### Latencies

Correct word responses with latencies between 150 and 1,500 ms were analysed. All word targets received at least 60% correct responses, our planned cut-off for analysis, so all word targets were retained.^9^ Mean latencies are shown in Table 2.

**Table 2:** Mean correct lexical decision times and lexical decision accuracy to words following insertion or deletion primes manipulating repeated or unique letter identities

	Insertion primes		Deletion primes	
Target	*Repeated*	*Unique*	*Repeated*	*Unique*
R**E**J**E**CTS	reject**c**s	reject**a**s	rjects	rejecs
MEA**N**I**N**G	m**a**eaning	m**c**eaning	meanig	maning
Response time (ms)	693	691	689	704
Accuracy (%)	94.6	93.7	93.8	93.9

*Note:* Bold font and underlining were not part of the stimuli but are shown here to highlight the relevant parts of the stimuli for the reader

The latencies were initially analysed with a Gaussian linear mixed-effect model (using the *lme4* package in R; R Core Team, 2023) with fixed effects for prime manipulation type (insertion vs. deletion), manipulated letter type (repeated vs. unique) and their interaction, and random components for subjects and targets that included intercepts and random slopes for all fixed effects, with random correlations.

To resolve singular fits, the following terms were dropped: all random correlations; the random slope on subjects for the main effect of repetition; and the random slope on targets for the interaction.

In Type III analysis of variance Wald tests (using the *car* package), neither main effect was significant: prime type (insertion vs. deletion), *χ*^2^(1) = 2.66, *p* =.103; letter type: *χ*^2^(1) = 2.56, *p* =.109. There was, however, a significant interaction, *χ*^2^(1) = 4.60, *p* =.032. Contrasts (using the *phia* package) showed significantly shorter response times for primes that deleted repeated letters than those that deleted unique letters, *χ*^2^(1) = 7.09, *p* =.008. There was no corresponding difference for primes that inserted either repeated or new letter identities, *χ*^2^(1) = 0.28, *p* =.600.

#### Accuracy

Accuracy was analysed for trials with latencies between 150 and 1,500 ms, as summarized by condition in Table 2. A binomial mixed-effect model was used, in which random correlations were excluded to avoid singular fit. None of the fixed effects was significant, all *p*s ≥.3.

## Discussion

For insertions, Experiment 1 replicated the results of Van Assche and Grainger’s (2006) Experiment 1: There was no difference between the insertion into the prime of a repetition of an existing letter and a new letter. There was no support for Adelman’s (2011) and Norris et al.’s (2010) interpretation (informed by a trend in Van Assche and Grainger’s Experiment 2) that the effect was absent due to lack of power.

For deletions, the result that a prime constructed by deleting a letter led to shorter response times when that letter was a repetition than when it was unique was consistent with non-significant trends in prior studies, suggesting those results were due to a lack of power. Schoonbaert and Grainger’s (2004) Experiment 1 had essentially the same manipulation and showed a 7-ms trend. Norris et al.’s Experiment 3b used a between-targets comparison, so the non-significant 11-ms interaction (8-ms vs. 19-ms priming vs. the identity control) is the relevant comparison.

The overall pattern is the opposite to that found by Trifonova and Adelman (2022) in the same-different task and is contrary to the LTRS predictions. The leakage and bigram models^10^ discussed in the *Introduction* predict a variety of different patterns that do not match the data. Experiment 2 tested whether the irrelevance of the difference between repeated and unique insertions is a general feature of the lexical decision task.

## Experiment 2

We examined response times to nonwords that served as primes in Experiment 1 and were constructed from their base words by inserting or deleting letters that were either repetitions or unique.

### Method

#### Participants

One hundred and sixty-two first-year undergraduates participated for partial course credit. Data for non-native speakers of English (52) were removed. The nonwords were much more word-like than those of Experiment 1, because they were our stimuli of interest, and so a lower accuracy threshold of 70% was used, which removed 13 participants. A further nine – the latest collected in their lists – were removed to ensure equal numbers in counterbalancing lists. So, the data of 88 participants were analysed.

#### Stimuli

For the nonword trials, the targets were the constructed nonword stimuli described in the *Common stimuli of interest* section. The base words were not presented.

Additional filler word trials were constructed using 144 new words from the British Lexicon Project with the same distribution of (letter) lengths as the nonwords. Three-quarters of these words had at least one non-adjacent letter repetition, to resemble the nonwords, and all had an empirical accuracy of at least 85% in that database.

#### Counterbalancing lists

Four counterbalancing lists were produced, each containing 36 of each of the four types of constructed stimuli. Each list contained one item constructed from each base word such that every constructed item appeared exactly once across the lists. All lists used all 144 filler words.

#### Procedure

Participants were instructed that their task was to examine the upper-case letter string on each trial and press the M key if it was a real English word or the Z key if it was not. Following 20 practice trials, all trials from the relevant counterbalancing list were presented in a new random order.

On each trial, a PsychoJS (Pierce et al., 2019) server showed the target in Courier New at 10% of display height until response. Feedback was given after incorrect responses only.

### Results

#### Latencies

Correct nonword responses with latencies between 150 and 1,500 ms were analysed for latency. Mean latencies are shown in Table 3.

**Table 3:** Mean correct rejection response times and accuracy in lexical decision to nonwords constructed by insertion or deletion of repeated or unique letter identities

	Insertion nonwords		Deletion nonwords	
Base word, e.g.,	*Repeated*	*Unique*	*Repeated*	*Unique*
R**E**J**E**CTS	reject**c**s	reject**a**s	rjects	rejecs
MEA**N**I**N**G	m**a**eaning	m**c**eaning	meanig	maning
Response time (ms)	922	865	849	851
Accuracy (%)	69.0	93.7	75.7	87.8

*Note:* Bold font and underlining were not part of the stimuli but are shown here to highlight the relevant parts of the stimuli for the reader

The latencies were analysed analogously to those in Experiment 1; only random correlations were dropped to avoid a singular fit.

The Type III analysis of variance Wald tests showed two significant main effects and a significant interaction: insertions versus deletions: *χ*^2^(1) = 33.08; unique versus repeated: *χ*^2^(1) = 31.99; interaction: *χ*^2^(1) = 13.41, all *p*s <.001. Contrasts (using the *phia* package) showed significantly longer response times for nonwords that inserted repeated letters of their base word than those that inserted new letters, *χ*^2^(1) = 41.44, *p* <.001. There was no corresponding difference for nonword targets that deleted either repeated or unique letter identities, *χ*^2^(1) = 0.77, *p* =.380.

#### Accuracy

Mean accuracies by condition are shown in Table 3. Response accuracy was analysed with a binomial linear mixed-effect model with logit link, analogous to the response time model. Manipulations involving unique letters yielded greater accuracy than those involving repeated letters, *χ*^2^(1) = 201.3, *p* <.001. Insertions and deletions did not, on average, differ significantly, *χ*^2^(1) = 3.11, *p* =.078. There was a significant interaction such that the impact of repetition on accuracy was greater for insertions, *χ*^2^(1) = 29.17, *p* <.001. The interaction was ordinal: Manipulations involving repetitions yielded lower accuracy for both insertions, *χ*^2^(1) = 170.0, *p* <.001, and deletions, *χ*^2^(1) = 31.96, *p* <.001.

### Discussion

When nonword stimuli are effective primes for target words, the fact that they seem to access those targets suggests that when used as foils in a lexical decision, they should be hard to distinguish from words. As such, we expect the response time pattern to be reversed, with manipulations of repeated letters leading to delayed responses. Manipulations of repeated letters led to slower responding, but only for insertions, not deletions. The error rates, however, painted a different picture: Stimuli created by insertion and by deletion of a repeated letter were less accurately rejected than stimuli created by analogous manipulations with unique letters, though this effect was stronger for the insertions.

The results tend to confirm the importance of the distinction between repeated and unique letters for processing in lexical decision, including when the repeated letter is inserted.

## Experiment 3

Experiment 3 was the primed same-different experiment. We examined response times on same trials to words that both preceded and followed primes with inserted or deleted letters that were either repetitions or unique. The change of task was used to investigate whether the inconsistency between the priming results of the lexical decision task of Experiment 1 and the same-different task of Trifonova and Adelman (2022; Experiments 2 and 3) could be primarily attributed to differences in the stimuli such as lexicality, or to the difference of task.

### Method

#### Participants

Seventy-four first-year undergraduates reporting English as their native language^11^ participated for partial course credit. Two were removed for accuracy below 85%. Four more – the latest collected in their list – were removed to equalize numbers in counterbalancing lists, leaving 68 for analysis.

#### Stimuli

For each of the same trials, the stimuli were the *Common stimuli of interest* given above: The primes were the nonword stimuli and the targets and references (which were the same) were the base word stimuli described there.

For the different trials, new reference-target pairs were selected from SUBTLEX-UK in which the reference and target differed by a single letter (i.e., were Coltheart neighbors). All references and targets contained a repetition with a single intervening letter that did not involve an exterior letter. The number of stimuli of each length (in letters) matched that of the base words. Primes for these trials were constructed based on the reference stimuli (not the targets), with each reference having all four types of prime. This was similar to the same trials, but without the pairing of items to match positions and identities of manipulations, and some items had insertions at the end, rather than internally.

#### Counterbalancing lists

Four lists were constructed that each had a quarter of trials with each prime type split evenly across same and different trials. Across lists, every designed reference-prime-target combination appeared exactly once.

#### Procedure

Participants were instructed that their task was to compare the first (lower-case) and final (upper-case) stimulus on each trial and press the right shift key if they were the same (ignoring the differences in case) or the left shift key if they were not. Following eight practice trials, all trials from the relevant counterbalancing list were presented in a new random order.

On each trial, DMDX (Forster & Forster, 2003) displayed the ########## mask for 1 s, then the 20 pt Courier New lower-case reference for 1 s, then the 12.5 pt lower-case prime for 60 ms, then the 20 pt upper-case target until response or a maximum of 3,000 ms had elapsed. Feedback was given after incorrect responses only.

### Results

#### Latencies

Correct ‘same’ responses^12^ with latencies between 150 and 1,500 ms were analysed. Mean latencies are shown in Table 4. These latencies were analysed analogously to those in Experiment 1, except that to avoid singular fits for this data set, we dropped the random slope for the interaction varying by subjects, and dropped all random slopes by targets. In Type III analysis of variance Wald tests, deletion primes were associated with significantly shorter response times than insertion primes, *χ*^2^(1) = 6.06, *p* =.014, with no significant difference overall between manipulations of repeated and unique letters, *χ*^2^(1) = 1.78, *p* = 1.83. A significant interaction qualified these effects, *χ*^2^(1) = 4.54, *p* =.033. Contrasts showed significantly shorter response times for primes that inserted repeated letters than those that inserted new letters, *χ*^2^(1) = 5.76, *p* =.016. There was no corresponding difference for primes that deleted either repeated or unique letter identities, *χ*^2^(1) = 0.15, *p* =.697.

**Table 4:** Mean correct same response times and accuracy on same trials in the same-different task to word after insertion or deletion primes manipulating repeated or unique letter identities

	Insertion primes		Deletion primes	
Reference/target, e.g.,	*Repeated*	*Unique*	*Repeated*	*Unique*
R**E**J**E**CTS	reject**c**s	reject**a**s	rjects	rejecs
MEA**N**I**N**G	m**a**eaning	m**c**eaning	meanig	maning
Response time (ms)	614	626	612	610
Accuracy (%)	95.0	94.8	95.5	95.3

*Note:* Bold font and underlining were not part of the stimuli but are shown here to highlight the relevant parts of the stimuli for the reader

#### Accuracy

Accuracy was analysed for trials in which the target and reference matched with response times between 150 and 1,500 ms. A binomial mixed-effects model was used with no random correlations; random slopes for both main effects for subjects and for the main effect manipulation type was also dropped for targets. There were no significant fixed effects, all *p*s >.24.

## General discussion

When manipulations involving repeated or unique letters are compared, and there is a difference between these manipulations, it is always in the direction that manipulating repeated letters results in a stimulus that is more confusable with the original than the manipulation of a unique letter. This suggests that the repeated letter manipulation creates a stimulus that is more similar to the target or base word than the stimulus with the unique letter manipulation. These findings are problematic for theories suggesting the same processing mechanisms for repeated and unique letters. However, these repeated letter effects did not always emerge for both insertions and deletions of repeated and unique letters, varying by task and measure.

Across three experiments, we used the same stimuli inserting and deleting repeated or unique letters to English base words. In masked primed lexical decision (Experiment 1), only using these stimuli as deletion primes, not as insertion primes, showed a repetition effect. In unprimed lexical decision (Experiment 2), using these stimuli as lexical decision foils, repetition had an effect on accuracy for both the stimuli with inserted letters and those with deleted letters relative to their base words, but it had an effect on response times only for the stimuli with inserted letters. In the masked primed same-different condition (Experiment 3), there were again asymmetrical effects of repetition, this time such that only insertion primes showed a repetition response time advantage, but no repetition effect for deletion primes.^13^

### Primed lexical decision

The results for insertion primes in lexical decision in Experiment 1 were consistent with the prior report of Van Assche and Grainger (2006) that primes constructed by insertions of repeated and unique letters do not differ in facilitation. The results for deletion primes, though, did show a significant difference, with those omitting a repeated letter showing facilitation relative to those omitting a unique letter. This effect was inconsistent with the null result of Schoonbaert and Grainger (2004), though across two prior reports, the deletion primes showed a numerically greater repetition effect: Norris et al. (2010) had concluded from their Experiment 3b that there was a repetition effect for deletion primes, although they did not have evidence for this effect from a significant interaction.

### Primed same-different task

The primed same-different results of Experiment 3 with words matched those of Trifonova and Adelman (2022; Experiments 2 and 3) with nonwords and the predictions of LTRS. Primes constructed by insertion of a repetition of an existing letter were associated with shorter response times than those constructed by insertion of a new unique letter, consistent with many models.

Primes constructed by deletion were equivalent for deletions of repeated and unique letters. In terms of the repetition effect in deletions, the prior report of such an effect by Norris et al. (2010; Experiment 3a) relied on the comparison of a significant effect and a non-significant effect but there was no statistical support for these effects being different: the interaction was not significant. Moreover, Norris et al.’s experiment concerned adjacent repetitions and shorter words. Among models that can predict repetition effects in other conditions, LTRS is the only one that predicts the absence of a repetition effect for deletion primes.

### Nonword rejections in lexical decision

When considering the primes of the other experiments used as targets for rejection in lexical decision in Experiment 2, the effects in correct response times were conceptually consistent with the primed same-different task, not the primed lexical-decision task: There was only a repetition effect for insertions. The accuracy data, however, showed effects for both insertions and deletions, though they were much smaller for deletions.

Both results for insertions are consistent with those of Kerr et al. (2021). The results for deletions were not entirely consistent with those of Schoonbaert and Grainger (2004; Experiment 2), who found no difference between the repeated and unique deletion conditions in both reaction times and accuracy. In the present Experiment 2, even though the reaction times were similar, participants were significantly less accurate in the repeated condition than in the unique condition.

### How can task dependency be accounted for?

The differences in results between tasks pose a substantial challenge for our understanding of priming as a means of investigating orthographic representations and processing. They suggest that priming results should not simply be interpreted as the degree of overlap between the representations of the prime and the target without any consideration of the task and the paradigm involved. The reversed pattern of the masked-priming results in the present studies was not entirely unexpected. The same-different task pattern replicated the previous findings of Trifonova and Adelman (2022), demonstrating repeated letter effects with insertions but not deletions. Previous masked priming lexical-decision task results showed no effects with both insertions and deletions, but the repeated letter effect with deletions observed here might be enhanced due to the increased power provided by greater numbers of subjects and trials.

Unlike some prior studies with letter repetition, the present results are not particularly susceptible to the suggestion that all repetition effects are equal and that some effects are obscured by lack of power. The experiments we report are more powerful than the prior studies due to the greater numbers of subjects and trials, as reported in Table 1 (we estimate our power to detect a simple repetition effect of 12 ms to be approximately.79, based on prior priming data in our laboratory – and the effect was in the opposite direction in only 0.5% of simulations; see Supplementary Materials). Moreover, the results we have reported are broadly consistent with prior findings, in that the repetition effects we have found to be significant were ones that had larger effects in prior studies (albeit either non-significant or with nonword rather than word materials), and those we did not find significant were smaller (and also not significant) in prior studies. More importantly, even if there are some small repetition effects in the cases where we have not found them (i.e., we have Type II errors), the interactions in Experiments 1 and 3 are still in opposite directions (and significantly different; see Footnote 13), so that there is a difference [between tasks] that requires substantive explanation. It does not appear that the relevant explanation is that the primed same-different task being more sensitive (leading to more powerful tests) than the primed lexical decision task can account for the differences; the same-different task did not lead to a larger effect. Moreover, unlike comparisons across articles with different experiments, the present design rules out the possibility that these differences are due to the stimuli of interest, rather than (some aspect of) the task, because the stimuli were re-used.

Therefore, the present findings suggest that the problem of repeated letters is important for the understanding of orthographic processing in terms of both lexical access and letter position and identity encoding. They also suggest that the choice of the task can have a strong effect on the observed results and should be taken into consideration for the interpretation of the results. As such, it is worthwhile to consider more mechanistic explanations where different tasks engage various representations or processes to different extents, or indeed different representations or processes, that make the different tasks more or less sensitive to insertions and deletions. There are many examples of models that have different representations at different levels, at different time points, or in different processes of the model. For instance, in order to account for relative position and transposed-letter effects, many models posit an additional representation that intercedes between a positional map like that of the McClelland and Rumelhart (1981) model and the lexicon. There is little reason to suppose that the brain has only one neural representation of strings. Any of these types of differences might underly differences between tasks in the effects shown. Indeed, the same-different task requires a comparison of the percept with a short-term memory representation, while the lexical decision task requires comparisons with lexical entries; although short-term traces inform long-term storage, they are clearly not identical. Generally, when there are different representations or processes at different levels or stages of the model, there is scope for different tasks to more strongly measure representations and processes at these different levels or stages.

#### Task demands

In terms of task demands, the lexical decision task more clearly places demands on lexical access than does the same-different task; the longer response times for lexical decision likely reflect, at least in part, the additional demands on lexical access. Lexical access, and hence lexical decision, may rely especially on letter identity information, especially during early processing from a briefly presented prime, as access is content addressed; as such, in lexical decision, missing information from unique deletion primes would be especially disruptive to access compared to primes containing all relevant letter identities. By contrast, in the same-different task, the task set is a comparison to the reference stimulus that requires the detection of inconsistent information, of which a novel letter would be the most salient instance, leading to disruptions of ‘same’ responses especially for unique insertion primes. As such, the success of LTRS in explaining the same-different task results might reflect the fact that the LTRS processes are primarily based on detecting inconsistent information.

#### Dimensional weighting

One version of the above explanation is that different dimensions of the same representation are more or less relevant in each task at a specific time. For example, perceiving that (all) the correct letter identities (or bigrams) are present in a stimulus might be more important for solving same-different and perceptual identification tasks than the primed lexical decision task. Perceiving all correct letter identities (even in duplicate) may also lead to rejecting nonwords being especially hard (as in the current Experiment 2). This pattern of differential weightings would be consistent with evidence from same-different tasks with varying methodologies suggesting that repeated letters have a facilitation effect for same trials and an inhibitory effect for different trials (Mirault et al., 2022; Trifonova & Adelman, 2022, Experiment 4), implying that the increased likelihood of perceiving a correct identity biases the decision towards ‘yes’. It is also consistent with the results showing that foils with repeated letter insertions are harder to reject than unique letter insertions (Trifonova & Adelman, 2022, Experiment 1) and the stronger facilitation priming effect with repeated insertion primes in same-different tasks (Trifonova & Adelman, 2022, Experiment 3; the present Experiment 3). The lack of evidence for greater priming with repeated letter insertions than unique insertions in the primed lexical decision task (the present Experiment 1) might be due to a relatively lower priority for perceiving the presence of all correct identities in very early stages. One reason for a lower priority could be a process of initially assessing the prime as a word at a more holistic level. The fact that the repeated letter manipulation led to significant differences with deletions and not insertions in Experiment 1 could be connected to the shorter deletion primes being more word-like than the longer insertion primes, thus possibly allowing for other factors, such as number of unique identities, to have an effect as well. If such a more holistic process has higher priority for the primed lexical decision task than verifying that all correct identities for a particular lexical item (that is about to be accessed) are present, additional evidence for one correct identity in the insertion condition might have less importance in the less word-like insertion stimuli.

#### Changes in representation over time

An alternative version of the explanation is that the representation changes qualitatively, adding new dimensions of information (rather than strength of evidence increasing as the representation is clarified, with (average) ranking preserved) This idea was explicit in Adelman’s (2011) LTRS model and was foreseen by Grainger et al. (2006) as a consequence of changes in positional uncertainty.

In the case of the LTRS model, there are differences in early processing between repeated and unique manipulations only for the insertion primes, and these are later resolved into perfect representations; an additional process would be needed for differences to occur between deletion primes. It might be natural to suggest that an additional process occurs on the final perfect representations. It is unclear, though, how a later process could be relevant for the failure of LTRS to predict the pattern of response times in primed lexical decision, as this complete representation is rarely reached during the prime, and as the process would need to be specific to this task.

In contrast, the approach of Grainger et al. (2006) and Grainger and Ziegler (2011) argues that an approximate code – such as open bigrams – enables efficient access to the lexicon. This approach builds on the general approach (e.g., Morton, 1964) of content-addressable access to the lexicon in which positive evidence is critical for activation (different accounts in this tradition give negative evidence different relative strength). The primed lexical decision results of the current Experiment 1 could be accounted for by only the difference in number of positively activated bigrams (i.e., neglecting inconsistent bigrams). Thus, the present range of results would be explained by negative evidence affecting performance only later – or when a reference stimulus is established in the same-different task – when a more precise code is in use.

However, other results in the primed lexical decision task speak against an approach purely based on positive evidence. For instance, Lupker et al. (2020; replicating a result of Adelman et al., 2014, but with length-specific controls) found that prefixing a letter (e.g., zjudge – JUDGE) and replacing the first letter (e.g., zudge – JUDGE) produced equivalent priming, despite the additional positive evidence when the first letter is not deleted.

#### Different representations in short-term memory versus the lexicon

The representations involved in the two tasks may qualitatively differ not because of timing but because of the nature of the required comparison. Were we to consider only Experiments 1 and 3, it might be plausible to suggest that these differ in which mode of representation – short-term or long-term lexical – is being accessed: Lexical decision compares the stimulus to a long-term lexical representation, whereas the same-different task involves a comparison to a representation in short-term memory. Indeed, one of the reasons that it is not straightforward to apply many models to the same-different task is because their matching process derives from permanent connections between the lexicon and lower levels, rather than an abstract computational system in which data and process can be separated. While Norris and Kinoshita (2008; Kinoshita & Norris, 2009; Norris et al., 2010) have argued that this representation is more raw and orthographically focused than the lexical representation used in lexical decision, Lupker and colleagues (Lupker, Nakayama et al., 2015a, 2018) have found evidence of phonological influences in the same-different task. Short-term representations of verbal material – which are needed to perform the same-different task – would be expected to exist in phonological as well as visual storage in short-term memory; it is not clear whether or how these would influence priming, but there is little reason to expect it to be similar to the influence of lexical phonology from lexical memory. Other ways in which there may be a greater lexical involvement in lexical decision than same-different could be online as in lexical competition (e.g., Lupker & Davis, 2009) or offline, reflecting the learned associations between orthographic and lexical representations (Adelman & Trifonova, 2022).

However, distinguishing between the representations in same-different and lexical decision tasks cannot explain why the unprimed lexical decision of Experiment 2 has the same pattern of results – at least in response times – as primed same-different and not primed lexical decision. Any version of the argument that the relevant difference is that primed lexical decision involves more lexical processing than primed same-different would still need to involve additional mechanisms sensitive to repetition with countermanding influences to explain the observed patterns in lexical decision without primes.

#### Stimulus sequence

An alternative analysis focuses on the stimulus sequence. Different paradigms differ not only in the goal of the tasks but also in the order of the perceived events. In the primed lexical decision task of Experiment 1, when the deletion primes are presented, the first displayed stimulus is a shorter stimulus, the prime, and then a longer stimulus, the target, must be processed. In the prime same-different task of Experiment 3, when the insertion primes are presented on same trials, a shorter stimulus, the reference (target) is presented first, and then the longer prime must be processed. So, this is something that conditions that show a repetition effect have in common. The same description applies to Experiment 1 of Trifonova and Adelman (2022), in which participants identified briefly presented strings in a two-alternative forced-choice paradigm: The repetition effect occurred for short targets that subsequently were compared to longer foils (as well as the correct alternative). In the analysis based on stimulus sequences, the present Experiment 2 is entirely different, because each trial has only one stimulus, and the final stimulus that participants see differs between conditions, neither of which is true of the other experiments. Otherwise, the effects only occur when the change from first to second stimulus is from shorter to longer.

If the stimulus sequence is the relevant cause of differences between the experiments, then a process of updating the abstract orthographic representation could occur as a result of each stimulus change. To explain the results with this type of mechanism, the assumption would be needed that once an initial stimulus representation is established, adding a new identity generates more processing dysfluency than adding a repetition. This could be true either because the change that adds the repetition is harder to notice, for instance, due to masking (Sayim & Taylor, 2019), or because adding a new identity is a more substantial change to the representation. By contrast, the loss of a letter can be handled fluently (and may be disregarded as potentially the result of an incomplete percept), regardless of repetition.

The stimulus-change mechanism accounts for the results in the primed lexical decision because the target follows the prime (and so the condition names do not match the temporal order). For the deletion primes, the change from prime to target involves *adding* a letter to the stimulus, requiring the type of representational update that differs between repetitions and novel letters; the difficulty of updating the representation to accommodate a new identity slows responding, either because target processing is delayed or because the difficulty disrupts the ‘familiarity’ signal. For the insertion primes, the change from prime to target involves a letter becoming absent from the stimulus; this representational update can be presumed to be similar in its effect between repeated and unique letters.

The stimulus sequence and patten of results is similar in the forced-choice identification task of Trifonova and Adelman’s (2022) Experiment 1. If the stimulus change mechanism is relevant, it is when the brief target stimulus has disappeared and there is an attempt to establish a representation of the foil. If establishing that representation is difficult, that would suggest that the new stimulus is different, causing greater accuracy. When the later stimulus – now the foil – is longer, then the addition of a new letter identity causes dysfluency. In this case, this increases accuracy, because it is a clue to the participant that there is a difference.

In the primed same-different condition, the trials of interest all involve both the change from shorter to longer stimulus and the one from longer to shorter stimulus, with the ordering differing between insertion and deletion primes. For the stimulus-change mechanism to account for a repetition effect only in insertions, it must be the *first* change – from reference to prime – that is relevant, because then insertion primes, which are the ones that show an effect, are associated with a change from shorter to longer stimuli – that is, actually an addition, as the name suggests.

## Conclusion

What is clear is that current models do not have the capacity to account for the differences we have observed between priming in lexical decision and same-different tasks. Broadly speaking, it has been necessary to assume that models will perform essentially similarly between different tasks, because the primed same-different task is rarely modeled, and when it is, essentially the same low-level mechanisms are assumed to be relevant as for primed lexical decision. Clearly, our experiments suggest that this assumption is unwarranted in at least some respect. Certainly, it is not new to suggest that processes outside of orthographic matching may influence the results of priming studies, nor that different tasks will give different results (e.g., Kinoshita & Norris, 2009, 2012), but the present results do not suggest that the difference is susceptible to the same explanations as task differences in lexicality effects, nor by simply overall sensitivity or power. Rather, the different tasks are sensitive to different manipulations of repetition: That is, the repeated letter effects were observed in both tasks, but only in deletions in the lexical decision task and only in insertions in the same-different task, so it is not the case that only one of the tasks is sensitive enough to detect the effect. A mechanistic account of the difference between tasks is required, which may take one of many forms. The tasks have different processing demands and may emphasise different components of processing. They may weigh different representational dimensions differently or involve different representations as representations change over time, or because of differences between short-term memorial and long-term lexical representations. Alternatively, different stimulus sequences may involve different relationships between stimulus changes and representational updates. In any case, any current model would require a substantive addition or modification to fully implement the differences between tasks. The present evidence regarding effects involving repeated compared with unique letter places will inform approaches to the task-specific aspects of studies of visual word recognition, and their relation to reading more broadly.

## Data Availability

All materials, data and analysis code are provided on the OSF at https://osf.io/a43h7/

## Footnotes

1 However, it is not clear that this argument is correct, because there is an inconsistency in the counting in the balace-BALANCE list, as AC is counted twice, but BA is not, despite both occurring twice in balace (once adjacent, and once with two intervening letters). Moreover, Schoonbaert and Grainger argue that the difference in number of *unique* bigrams allows the model to predict longer response times for targets with repeated letters (fewer unique bigrams) than those without, suggesting that only unique bigrams should be counted. That is, both AC and BA should be counted only once, giving only nine matching bigrams, which would mean “balnce” should be a stronger prime than “balace” (and if they are to be counted twice, then “balace” would have eleven consistent bigrams and be the stronger prime). Applying this unique bigrams rule (i.e., without double-counting), then, the model predicts a repetition effect for deletions such that deleting a repetition should produce stronger priming than deleting a unique letter (or the reverse prediction if the double-counting is applied consistently).

2 This argument about consistent bigrams only applies to stimuli like van Assche and Grainger’s (2006), which inserted letters at least two positions away from the end of the targets. Nevertheless, all types of primes with repeated letter insertions are also predicted to be stronger primes than primes with unique letter insertions if one also considers the inhibitory role of inconsistent bigrams. (The role of inconsistent bigrams is often neglected in considering the predictions of open bigram models, because in many cases, they are simply the complement of the consistent bigrams, but that logic does not apply to repeated insertions.) Inserting a unique letter creates more inconsistent bigrams than inserting a repeated letter, and so there is an additional inhibitory influence for unique than repeated insertions, so the repeated letter prime should net more facilitation than the unique insertion prime. This difference in inhibition from inconsistent bigrams between repeated and unique insertions applies even when the number of consistent bigrams is the same (which occurs when the insertion is at, or next to, the first or final letter).

3 Moreover, this model with standard parameters predicts no meaningful repetition effect for the present experiments (max. 2 ms in Experiment 1 RTs; max 4 ms in Experiment 2. Response times (RTs); max. 1.4% in Experiment 2 accuracy).

4 It is not clear, however, that this argument is correct for their model: Norris et al. justify their argument with reference to a sloppy slots model discussed and rejected by Davis (2006). However, the principles of these models are not analogous: Davis’s (2006) argument that sloppy slots make the prediction of more priming from a repeated deletion than a unique deletion relies on the assumption that the two As of a “balace” prime provide no more activation than any other two letters, despite matching two As in BALANCE (this contrasts with the assumption, also necessary, that the single A of a “balnce” prime provides more activation than other single letters because it matches both As). Norris et al.’s model does not have this assumption: If it did, it would not predict a repetition effect for the substitution case of their Experiment 2.

5 This is manifestly a simplification in the model, and how best to weaken this assumption is an ongoing avenue of research.

6 The erroneous stimuli all come in the By Deletion nonwords: They were “flings” from F**I**LINGS; “statin” from STATI**O**N; and “catered” from CA**N**TERED. For Experiments 1 and 3, all planned trials involving these three base words were presented to participants but excluded from analysis (they were still presented in Experiment 3 for consistency with Experiment 1, though the error had been noted by this point). For Experiment 2, all stimuli constructed from those base words were presented to participants but excluded from analysis. Only in Experiment 2 did the erroneous stimuli require a response from participants (rather than being primes), and in this experiment, correct feedback (i.e., a nonword response was treated as an error) was given for these three words.

7 A reviewer also asked about the pronounceability and legality of these nonwords. Nonwords that were unpronounceable or had a clear orthotactic violation (according to the first author’s native speaker judgement) accounted for 84 out of 144 (58%) of repetition insertion stimuli and 74 out of 144 (51%) of unique insertion stimuli, *χ*^2^(1) = 1.14, *p* =.287; and for 44 out of 144 (31%) of the repetition deletion stimuli and 37 out of 144 (26%) of the unique deletion stimuli, *χ*^2^(1) = 0.62, *p* =.432, so again the differences between conditions were quite minimal.

8 Of those who completed Experiment 1, which was in-person, 101 did so before the Easter 2021 vacation, while 31 completed it in the two weeks after. Experiment 2 was made available online during that vacation, and 124 participants completed it during that vacation, but 37 did so in the two weeks after. In consequence, some (at most 31, but likely fewer) participants had already completed Experiment 2 when they participated in Experiment 1, but the other order was more common. Participants who completed Experiment 2 before Experiment 1 would have seen one-quarter of the Experiment 1 primes as lexical decision targets to be rejected in Experiment 2. Due to our counterbalancing scheme, for approximately a quarter of those participants, the primes of Experiment 1 would have been exactly the same as the nonword targets of Experiment 2. For the remainder, they would have been similar but not identical. Would seeing the nonword primes or nonword stimuli similar to them in as lexical decision targets in a different experiment in a different context at a different time lead to differential responding? The evidence regarding repetition effects for nonwords in lexical decision says not: Scarborough et al. (1977) found that such effects occur only for short lags within an experimental session (Experiment 1) and not at all across different experimental sessions (Experiment 4). Both were run under ethical approval for a general series of experiments, so we did not have ethical approval for de-anonymization to link the two data sets. The overlap with Experiment 2 came about as follows. We were obligated to provide another course credit opportunity to the cohort of Experiment 1 before a deadline that – in combination with laboratory social distancing – did not allow for in-person testing. We chose to run the unprimed lexical decision experiment before the same-different experiment (i.e., as Experiment 2 not 3) because our attempts to present 50- to 60-ms primes with PsychoJS resulted in primes that were, at times, clearly visible, suggesting that the control of prime duration was insufficient.

9 Analyses of nonword trials are available in the Electronic Supplementary Materials on the Open Science Framework (OSF) – neither latencies nor accuracies showed significant differences between conditions.

10 For instance, the constrained open bigram model predicts a repetition effect in deletions because of facilitation and in insertions because of inhibition. For deletions, rejcts-REJECTS has 10/13 facilitatory bigrams (EE, JE and ES [too distant] are missing) and one inhibitory bigram (JS) but rejecs-REJECTS has 9/13 facilitatory bigrams (JT, ET, CT and TS are missing) and one inhibitory bigram (JS). For insertions, rejectcs-REJECTS has two inhibitory bigrams (TC and CC) and 12/13 facilitatory (only ES is missing [too distant]), whereas rejectas-REJECTS has three inhibitory bigrams (EA, TA and AS) and 12/13 facilitatory (only ES is missing [too distant]).

11 Unlike the earlier experiments of this paper, non-native speakers were diverted to an alternative study.

12 Analysis of “different” trials is in the Electronic Supplementary Materials on the OSF. Neither latencies nor accuracy showed any significant effects of prime manipulations.

13 Given that the significant two-way interactions of insertion-deletion and repetition-unique in Experiment 1 and Experiment 3 have opposite signs, it is inevitable that the three-way interaction with task in a combined analysis is significant: *χ*^2^(1) = 8.95, *p* =.003. Further details of this analysis are available in the Electronic Supplementary Materials on the OSF.

## Code availability

All materials, data and analysis code are provided on the OSF at https://osf.io/a43h7/
